# Comparisons of the Effects of Polymer and Alcohol Varnishes on Norway Spruce Wood Surface Modifications

**DOI:** 10.3390/polym17152131

**Published:** 2025-08-01

**Authors:** Mariana Domnica Stanciu, Maria Cristina Timar, Mircea Mihalcica, Mihaela Cosnita, Florin Dinulică

**Affiliations:** 1 Department of Mechanical Engineering, Transilvania University of Brașov, B-dul Eroilor 29, 500036 Brasov, Romania; mihalcica.mircea@unitbv.ro; 2 Romanian Society of Rheology, Petru Poni Institute of Macromolecular Chemistry, Room 119, 41-A, Grigore Ghica Voda Alley, 700487 Iasi, Romania; 3 Romanian Society of Theoretical and Applied Mechanics, 266 Pantelimon, 2 Sector, Office 4, 021652 București, Romania; 4 Romanian Society of Acoustics, 266 Pantelimon, 2 Sector, Office 4, 021652 București, Romania; 5 Faculty of Furniture Design and Wood Engineering, Transilvania University of Brasov, 500036 Brasov, Romania; cristinatimar@unitbv.ro; 6 Department of Product Design Mechatronics and Environment, Transilvania University of Brasov, 29 Eroilor, 500036 Brasov, Romania; mihaela.cosnita@unitbv.ro; 7 Faculty of Silviculture and Forest Engineering, Transilvania University of Brasov, 1 Sirul Bethoven, 500123 Brasov, Romania; dinulica@unitbv.ro

**Keywords:** polymeric varnish for musical instruments, overall color change, surface morphology, Fourier transformed infra-red

## Abstract

Spruce wood is a natural polymeric material, consisting of cellulose, lignin, hemicelluloses and other secondary components, which gives it a unique chemical footprint and architecture. Varnishes are used in musical instruments to protect the wood against humidity variations, wood being a hygroscopic material, but also to protect the wood from dirt. The varnishes used both to protect the wood from resonance and to ensure a special aesthetic appearance are either polymeric varnishes (nitrocellulose, oil-based) or volatile solvents (spirit). In this study, the color changes, the surface morphology and the chemical spectrum produced by three types of varnishes, applied in 5, 10 and 15 layers, on resonance spruce plates were analyzed. The results revealed significant changes in the color parameters: the lightness decreased by approximately 17% after the first layer, by 50% after 5 layers, by 65% after 10 layers and by 70% after 15 layers. The color parameters are most influenced by the anatomical quality of spruce wood (annual ring width and earlywood/latewood ratio) in the case of oil-based varnishes and least influenced in the case of nitrocellulose varnishes. The chemical fingerprint was determined by FTIR spectrum analysis, which revealed that the most pronounced absorptions were the double band 2926–2858 cm^−1^, corresponding to aliphatic methylene and methyl groups (asymmetric and symmetrical C-H stretch), and the bands at 1724 cm^−1^ (oil-based varnish), 1722 cm^−1^ (nitrocellulose varnish) and 1708 cm^−1^ (spirit varnish), all assigned to non-conjugated carbonyl groups in either carboxylic acids, esters aldehydes or ketones. The novelty of the study lies in the comparative analysis of three types of varnishes used in the musical instrument industry, applied to samples of spruce resonance wood with different macroscopic characteristics in three different layer thicknesses.

## 1. Introduction

Norway spruce wood (*Picea abies* Karst. L.) is a natural polymer that, from the point of view of chemical architecture, contains three-dimensional polymer chains of carbohydrates, namely cellulose (50.69–50.90%), hemicellulose (pentoses (7.18–11.20%) and hexoses) and lignin (26.68–33.25%), as well as extractable substances and inorganic compounds [1,2,3,4]. The moisture absorption capacity of wood depends on the hydrophilic properties of the polymer chains of the main chemical constituents of wood, as well as on the accessibility of water to the polymer chains [5]. According to [5], the hydroxyl groups in hemicelluloses and lignin are easily accessible to water molecules due to their branched structure. Since wood is a hygroscopic material and exposed to dirt, various protective coatings have been used over time to ensure its aesthetic appearance and protect it from moisture. Researchers have long studied the chemical composition of varnishes used for historical musical instruments, with the aim of identifying the organic and inorganic compounds that could be responsible for the acoustic quality of musical instruments [6,7,8]. The results highlighted that the varnishes used are complex mixtures of organic (oils and resins) and inorganic (pigments, fillers, etc.) compounds, with different solvents/additives, such as essential oils, alcohol and oil-based varnishes [6,7,8]. According to the analysis made [7], the varnishes used for varnishing musical instruments, as coating substances that are applied in one or more layers, can be classified according to the solvent they contain in three categories: spirit-based; drying oil-based; essential oil-based. In the case of oil varnishes, the oil is vegetable oil; the most used are linseed oil, walnut oil and castor oil. Spirit varnishes are based on a mixture of resins dissolved in alcohol; that is why they are also called alcoholic varnishes. Resins are natural or artificial polymers having the property of softening or melting when the temperature gradually increases and can then be spread on support surfaces. Researchers classify natural resins produced by trees into two main groups: diterpenes (based on organic compounds of two terpene units C20H3) and triterpene resins (with three C30H48 terpenic units). Examples of diterpene resins include sandarac—the resin of the *Juniperus comunis* tree (northern Africa, Morocco), rosin—the resin of the species of the Pinaceae family, and copal—tree product from *Protium copal* (Burseraceae) (Mexico, Central America). Triterpene resins include dammar—*Canarium strictum* (India), mastic—*Pistacia lentiscus* tree (Grace), and elemi—produced by the *Canarium luzonicum* tree (Philippines). Besides these resins, fossil resins (amber), as well as resins produced by insects (shellac), gums, proteins, waxes and natural dyes, were also identified [7]. The varnishes were used to protect the wood against dirt, mechanical damage and humidity variations in the environment, as well as for the aesthetic values that the instrument acquired through color and gloss. Most studies have focused on the study of the chemical analysis of the different types of finishes used on historical instruments, color being one of the additional properties correlated with inorganic compounds [9,10,11]. Ref. [10] studied the nanomechanical properties of finishing samples using traditional violin varnish recipes applied to maple wood. The alcohol varnish, having a higher stiffness, penetrated the wood less than the oil-based varnish, and quantitative nanomechanical mapping demonstrated a difference in adhesion values between the oil-based and alcohol-based samples. Most studies have analyzed the relationship between the type of finish and the dynamic and acoustic properties induced by the stiffness of the new varnish–wood system, both immediately after the application and curing of the varnish [11,12,13,14] and after the application of aging treatments [15,16,17,18,19]. The physical properties that the lacquer for musical instruments must possess are gloss, transparency, color, wear, dirt resistance and adhesion to the wood, and among the mechanical properties, elasticity and the ability to improve the tone of the instrument. Varnishing soundboards with polymeric finishes or volatile solvents leads to changes in the rigidity and mass of the wood board, producing changes in its vibrational and acoustic properties [17]. Elasticity is the most important property of the lacquer since it must not affect the vibration mode of the lacquered instrument. Refs. [12,16,17,20,21,22,23,24] have demonstrated that varnishes increase the damping of wood along and perpendicular to the grain directions. The varnishes reduce sound radiation along the fiber but increase it in the perpendicular direction. However, there are few studies that analyze the modification of color parameters as a result of applying different types of varnishes to the resonance spruce wood. This paper aims to comparatively analyze the effect of varnish film thickness on the color parameters, surface morphology and chemical fingerprint of resonance spruce wood. The analysis was performed both before and after the application of each varnish layer, investigating three types of varnishes: spirit, oil-based and nitrocellulose. The novelty of the study lies in the quantitative and qualitative analysis of the color, surface morphology and chemical fingerprint of wood varnished with the varnishes used in the construction of stringed musical instruments.

## 2. Materials and Methods

### 2.1. Sample Preparation

**Figure 1:**
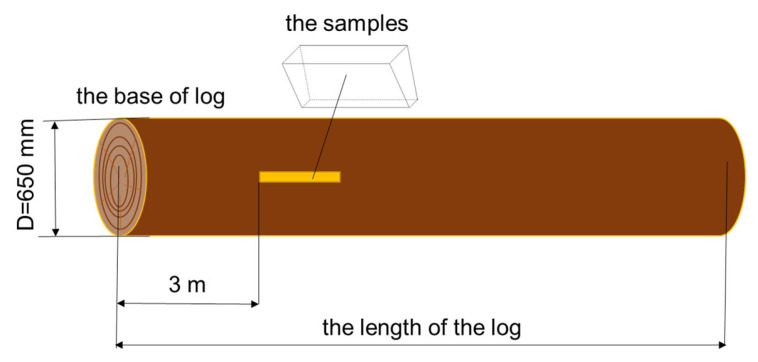
The position of the wooden blanks in the spruce logs.

**Figure 2:**
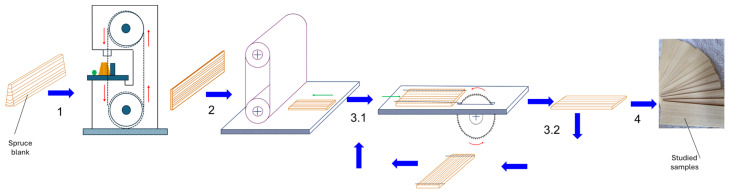
The main operations for sample preparation. Legend: 1—slitting of the blank on a band saw; 2—calibration of the faces on a sanding calibrating machine (four passes with different grits of the sanding roller); 3.1—straightening the edges for basing; 3.2—cutting (sectioning) to length; 4—the samples at the final dimensions. (Arrow legend and color code: Technological flow—blue arrow; part advance—green arrow; machine tool feed—red arrow).

The wood samples in the form of rectangular plates with dimensions of 200 mmx80 mmx4 mm were made of Norway spruce wood (*Picea abies* (L.) Karst.) harvested from the Romanian Carpathian mountains. The spruce trees were harvested from the Moldoviţa Forest District (Suceava—Bucovina), Romania, known for its resonant Norway spruce stands. The tree diameters were 600–650 mm, and the 4/4 violin blanks were extracted by quarter cutting at a base distance of approximately 3 m, as can be seen in Figure 1. The samples come from the raw material warehouse of the musical instrument factory, being prepared from semi-finished products for violins that have been naturally dried for 5–8 years, reaching a moisture content of 8–10%. The main technological operations involved in the preparation and obtaining of the samples, as mentioned in [25], are schematically presented in Figure 2.

Unlike the classification in STAS 10824-76 (Romanian standard) [26], where only two quality classes are mentioned for the faces/backs of musical instruments (A and B), each of which is divided into three subgroups, in this study, two types of sounding spruce wood used for violin faces are analyzed, having extreme anatomical characteristics. Class A designates the best quality of wood, and class D refers to the worst quality of wood used for musical instruments. The samples were grouped into two quality classes depending on the anatomical parameters, such as the width of the annual rings, the proportion of late wood/early wood, presence/absence of anatomical defects and regularity of annual rings. Grade A represents the samples with narrow annual ring widths, below 1 mm, regular, 74.97 ± 1.6% early wood proportion and 25.03 ± 1.5% late wood proportion and grade D represents the samples with annual ring widths greater than 2.5 mm, irregular, 76.36 ± 1.32% early wood proportion and 23.64 ± 1.13% late wood proportion [27,28]. Detailed information about the anatomical quality classes of sounding wood is detailed in the study previously presented in [27,28].

**Figure 3:**
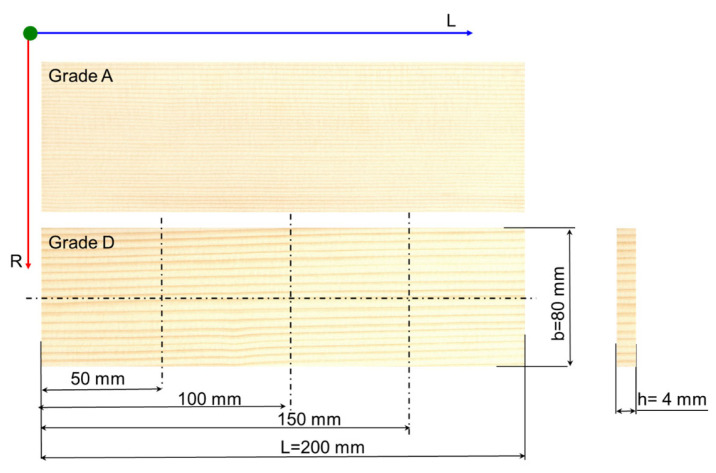
The aspect and sizes of samples from A and D quality grades of spruce wood (Legend: L—longitudinal direction of wood; R—radial direction of wood; L—length; b—width; h—thickness; 50 mm, 100 mm and 150 mm are the positions used for color measurement).

Figure 3 presents the two categories of spruce wood samples (coded with S). A total of 54 samples were prepared (27 from each quality class, 3 samples from each varnishing category). The marks positioned at 50, 100 and 150 mm represent the position of the wood color measurement points.

The whiteboards were subsequently grouped according to the type of varnish applied. These were grouped into three subcategories depending on the type of varnish, namely oil-based varnish coded LU, alcohol varnish coded LS and nitrocellulose varnish coded NC. All varnishes are commercial products, as can be seen in Table 1. Oil-based varnish (LU) contains a mixture of natural and synthetic resins dissolved in turpentine and linseed oil. Curing is based on the oxidative polymerization of oil. Nitrocellulose varnish (NC) is also an artificial polymeric product obtained from the treatment of natural polymeric cellulose with nitric acid, part of the hydroxyl groups in cellulose being replaced by nitrate groups. The solvent used contains ethyl acetate and butyl acetate, ketones such as acetone and methyl ethyl ketone, and aromatic hydrocarbons. Spirit varnish mainly uses ethyl alcohol (ethanol), which dissolves the resin, with shellac being used in the past. Currently, for musical instruments, various resins are used that have properties like shellac.

For each type of varnish, regardless of the substrate type A or D, three varnish thicknesses quantified from the point of view of the varnishing process were established depending on the total number of layers of finishing materials applied: 5, 10 and 15. It has to be specified that the technologies included a first phase of coloring with a water-based solution (layer 1 or layers 1–2), generally continued with application of several colored varnish layers, and ended with 3–5 layers of colorless varnish (without any coloring extract). As an exception, for NC finished samples, after the initial coloring, only colorless varnish was employed (layers 4–15). Also, the technologies were particularized for each type of varnish by the musical instrument manufacturer (Gliga Musical Instruments Factory), respecting the recipes and conditions related to the finishing of violins, as can be seen in Table 2.

**Table 2:** Outlook of the finishing technology with 15 layers for the three types of varnishes (Legend: KD—Kalium dichromate; WS—Water stains; WS-R—Water stains recipe; CEV—Color extracts for varnishes).

Layers	Spirit Varnish (LS)	Nitrocellulose Varnish (NC)	Oil-Based Varnish (LU)
1	KD	WS-125 yellow	WS-R
2	LS+CEV2901	WS-104 nut brown	LU
3	LS+CEV2901	NC base coat	LU+CEV2901
4	LS+CEV2903	NC	LU+CEV2901
5	LS+CEV2901+CEV2903	NC	LU+CEV2903
6	LS+CEV2901+CEV2903	NC	LU+CEV2903
7	LS+CEV2901+CEV2903	NC	LU+CEV2904
8	LS+CEV2904	NC	LU+CEV2904
9	LS+CEV2904	NC	LU+CEV2904
10	LS+CEV2904	NC	LU+CEV2904
11	LS	NC	LU+CEV2904
12	LS	NC	LU+CEV2904
13	LS	NC	LU
14	LS	NC	LU
15	LS	NC	LU

**Figure 4:**
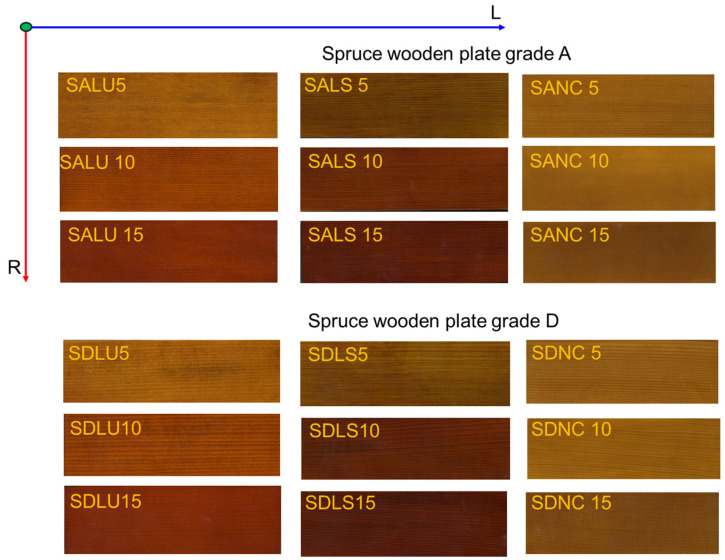
The varnished samples (Legend: SALU5—spruce wood grade A, varnished with oil-based varnish, 5 layers; SALS5—spruce wood grade A, varnished with spirit varnish, 5 layers; SANC5—spruce wood grade A, varnished with nitrocellulose varnish, 5 layers; SALU10—spruce wood grade A, varnished with oil-based varnish, 10 layers; SALS10—spruce wood grade A, varnished with spirit varnish, 10 layers; SANC10—spruce wood grade A, varnished with nitrocellulose varnish, 10 layers; SALU15—spruce wood grade A, varnished with oil-based varnish, 15 layers; SALS15—spruce wood grade A, varnished with spirit varnish, 15 layers; SANC15—spruce wood grade A, varnished with nitrocellulose varnish, 15 layers).

The general aspect of the various types of varnished samples can be viewed in Figure 4.

### 2.2. Methods

#### 2.2.1. Thickness of Varnishes’ Film

According to [12,14,29,30], to allow for a direct comparison between the mass changes of spruce boards varnished with different types of varnishes and with different film thicknesses, the changes were evaluated as an areal mass loading (denoted AML) induced by the coating system using relation (1).

(1) $$AML=\frac{m_{v}-m_{i}}{b\;*\;L},$$

where $m_{v}$ is the mass of varnished sample; $m_{i}$ is the mass of sample before varnish; b and L are the sizes of the sample. The mass was measured with a Kern DLB160-3A analytical balance (Kern&Sohn GmbH, Balingen, Germany).

To analyze the areal mass loading increase ∆AML of the samples because of the increase in the thickness of the varnish film, relationship (2) was applied:

(2) $$∆AML=\left(\frac{{AML}_{n+5}-{AML}_{n})}{{AML}_{n}}\right)\;*\;100,$$

where ${AML}_{n}$ is the AML value of samples with superior number of layers (n=5;10).

#### 2.2.2. Color Measurement

The first color measurements were performed before the varnishing process on all class A and D spruce wood samples, following the measurement scheme in Figure 3. The color parameters L* (lightness), a* (redness) and b* (yellowness) were measured with the colorimeter, using the CIE-L*a*b* color measurement system (according to CIE Commission Internationale de l’Eclairage), and based on them, E* was calculated [14,27,29,30,31]. The overall color change ∆E* was calculated with the following Equation (3):

(3) $$∆E*=\sqrt{{\left(∆L*\right)}^{2}+{\left(∆a*\right)}^{2}+{\left(∆b*\right)}^{2}},$$

where ∆L*, ∆a* and ∆b* are the differences between coordinates of the values L*, a* and b* of the varnished surface and before varnishing.

#### 2.2.3. The Contact Angle Method

**Figure 5:**
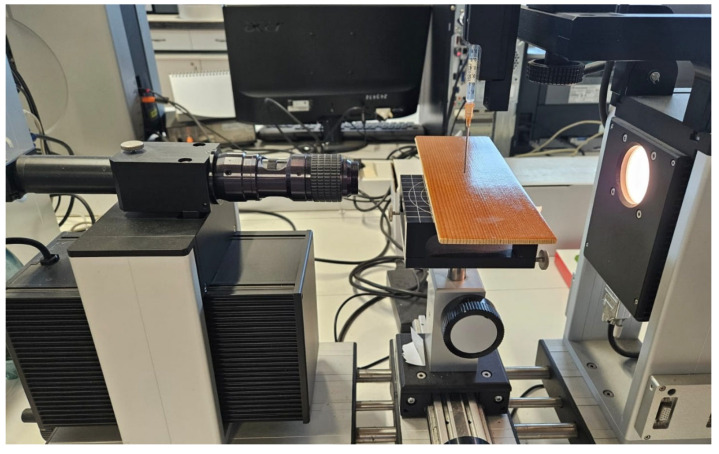
The testing of samples with System OCA-20 equipment.

The evaluation of the surface energy of the wood samples was carried out by the contact angle (CA) method. The value of the contact angle depends on three factors: the morphology of the substrate, expressed by the surface tension between the solid and gas medium (mN/m); the nature of the liquid, expressed by the superficial liquid and gas tension (mN/m); the nature of liquid and substrate interactions, expressed by the surface tension between the solid–liquid medium (mN/m) [32,33,34,35]. A liquid placed on a solid surface, in the absence of gravitational forces, will take the shape corresponding to the minimum energy of the system [36,37,38,39]. The CAs were determined with the System OCA-20 equipment (Data Physics Instruments, L7 Laboratory—ICD Transilvania University of Brasov) using successively distilled water and glycerin for the drop with a volume of 10 μL. Thus, the values of the contact angle for water and for glycerin were measured. The measurements were performed at room temperature and under normal humidity conditions (T = 22.7 °C and RH = 65%). Three measurements per sample were made in the same areas presented in the previous methods (Figure 5). Based on the images recorded every second, the dynamic studies of the contact angle changes allow the evaluation of the absorption/adsorption capacity of the samples. The CA can take values between 0 and 180°. If the CA < 90°, the analyzed surface is polar, i.e., the material is hydrophilic (absorbs liquid), and if CA > 90°, the surface is dispersive, i.e., the material has a hydrophobic character (does not absorb liquid). The type of interaction between that surface and the liquid used in the analysis can be estimated from the CA angle [37,38,39,40]. Surface energy is a quantity that characterizes the morphology of the surface, in its turn being responsible for the ability to absorb or reflect acoustic waves. The measurements were performed at the same points presented in Figure 3, positioned at 50, 100 and 150 mm from edge sample.

#### 2.2.4. Fourier Transformed Infrared (FTIR) Spectroscopy

Fourier transform infrared spectroscopy (FTIR) was used to analyze and compare the chemical characteristics of the untreated wood surface and the wood surfaces after coating with the three types of varnishes, applied in 5, 10 and 15 layers. FTIR analysis was performed with an Alpha Bruker spectrometer equipped with an attenuated total reflectance (ATR) unit. FTIR spectra were recorded in the range 4000–600 cm^−1^, at a resolution of 4 cm^−1^, 24 scans per spectrum, and processed with OPUS 7.2 software [33,34,35].

#### 2.2.5. Statistical Analysis

The statistical analysis consisted of data processing with STATISTICA 8.0 (StatSoft 2007) [36]. In the first stage, the sources of variation were probed, using the hypothesis of normal distribution of variables with the Shapiro–Wilk test; the significance or statistical relationship between variables was determined using Mann–Whitney U test and Kruskal–Wallis test, with *p*-values determining statistical significance. Subsequently, to rank the influence of various factors, such as the quality class of the wood used as substrate (denoted by SS), the type of varnish (TL) and the number of varnish layers (NL) on the studied properties, principal component analysis (PCA) was used. Finally, a multifactorial discriminant analysis of the measured properties, in relation to the variables mentioned above, was performed.

## 3. Results and Discussion

### 3.1. Variation in Areal Mass Loading

**Figure 6:**
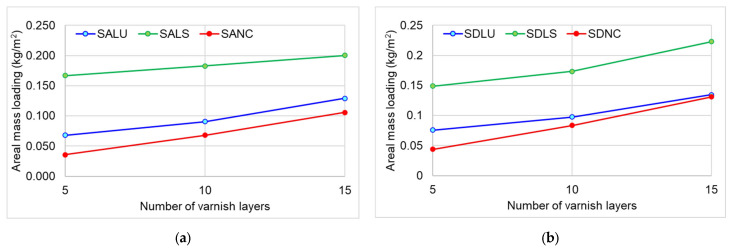
Variation in areal mass loading with type of varnish: (**a**) Grade A samples; (**b**) Grade D samples.

Figure 6 shows the variation in areal mass loading with increasing thickness of the varnish film. Thus, after the application and drying of the first layer of stain, it is sanded with 280 grit abrasive paper, and after the successive application and drying of the varnish films, they are sanded with 320 grit abrasive paper. The highest varnish consumption is recorded for spirit varnish (LS), approximately 2.4 times more than the surface consumption of oil-based varnish (LU), for five layers, for both class A and class D spruce wood. The lowest consumption is observed for the nitrocellulose varnish (NC), approximately 1.9 times less than the oil-based varnish (LU). With an increasing number of layers, areal mass loading increases regardless of the type of varnish, as can be seen in Table 3. The consumption of nitrocellulose lacquer is almost identical regardless of the quality of the wood. The lowest consumption of lacquer among the three categories of samples was recorded in the case of alcohol lacquer.

### 3.2. Variation in Color Parameters

#### 3.2.1. Changes in Color Parameters During the Varnishing Process

**Figure 7:**
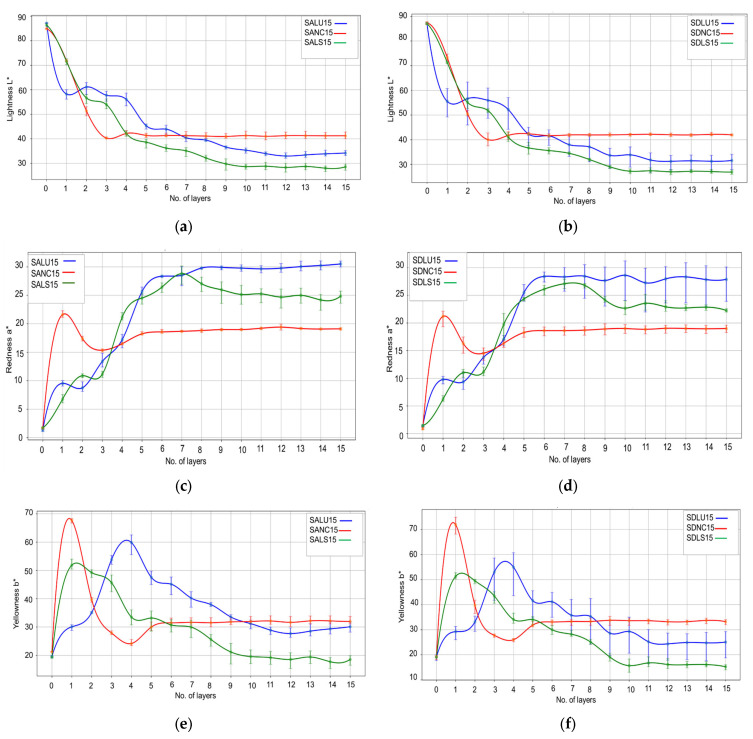
Changes in color parameters: (**a**) lightness L* for spruce wood grade A; (**b**) lightness L* for spruce wood grade D; (**c**) redness a* for spruce wood grade A; (**d**) redness a* for spruce wood grade D; (**e**) yellowness b* for spruce wood grade A; (**f**) yellowness b* for spruce wood grade D. Legend: SALU15, SDLU15—spruce wood grade A/D, varnished with oil-based varnish, 15 layers; SALS15, SDLS15—spruce wood grade A/D, varnished with spirit varnish, 15 layers; SANC15, SDNC15—spruce wood grade A/D, varnished with nitrocellulose varnish, 15 layers.

**Figure 8:**
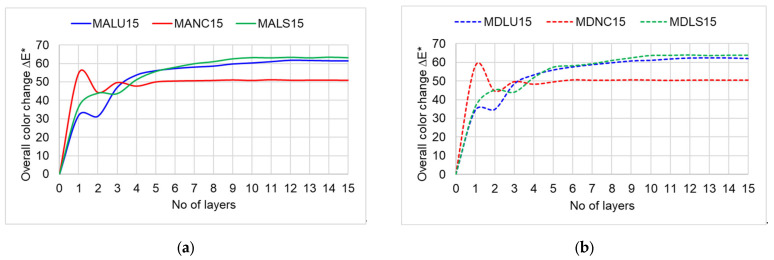
Overall changes in total color $∆E^{*}$. (**a**) for spruce wood grade A and (**b**) for spruce wood grade D. (Legend: SALU15, SDLU15—spruce wood grade A/D, varnished with oil-based varnish, 15 layers; SALS15, SDLS15—spruce wood grade A/D, varnished with spirit varnish, 15 layers; SANC15, SDNC15—spruce wood grade A/D, varnished with nitrocellulose varnish, 15 layers).

Figure 7 shows the evolution of the color parameters during the finishing process for the samples with 15 layers, for the three types of varnishes. In Figure 7, the layer marked with zero represents the initial wood substrate before varnishing, and the following corresponds to the successive layers of finishing materials, the color parameters being measured after the drying of each applied layer. In general, it is observed that the lightness decreased for all the samples after the staining phase, though the values differed as a function of the staining materials and the actual technology. For SALU15 and SDLU15 stained with WS-R (a mixture of red brown, pure yellow and nut brown dark), the decrease in lightness was 31–35% (layer 1). For the samples SALS15 and SDLS15 stained with KD, the decrease was only 16%. For samples SANC15 and SDNC15, with staining in two layers, the decrease was around 16% after layer 1 (WS-125 yellow) and reached about 40% after layer 2 (WS-104 nut brown) (Figure 7a,b). Further lacquering of these samples brought about a decrease in lightness only after the first applied layer, followed by stabilization throughout the rest of the finishing technology. For the samples SALS15 and SDLS15, as well as SALU15 and SDLU15, there was a wavy decrease of lightness up to layer 9 (varnishes colored with the darkest color extract), followed by stabilization. The evolution curves for the chromatic color parameters, redness (a*) and yellowness (b*), presented in Figure 7c–f are specific to the technology presented in Table 2. The most pronounced changes occur in the case of redness and yellowness, the parameters that increase by 300% (redness) and 600% (yellowness) after the application of the first layer. In Figure 8, an increase of 30–40% in the overall color can be noticed compared to the initial color of wood in white. The greatest overall color difference was obtained after applying the first layer of nitrocellulose varnish ($∆E^{*}=50\;÷60)$ compared to the LU and LS varnish, where ($∆E^{*}=30\;÷40)$. Starting with the fifth layer, the color difference remains approximately constant for all types of finishes analyzed, regardless of the quality of the substrate or the macrostructural particularities of the spruce wood.

#### 3.2.2. Changes in Color Parameters in Accordance with the Thickness of Varnish

**Figure 9:**
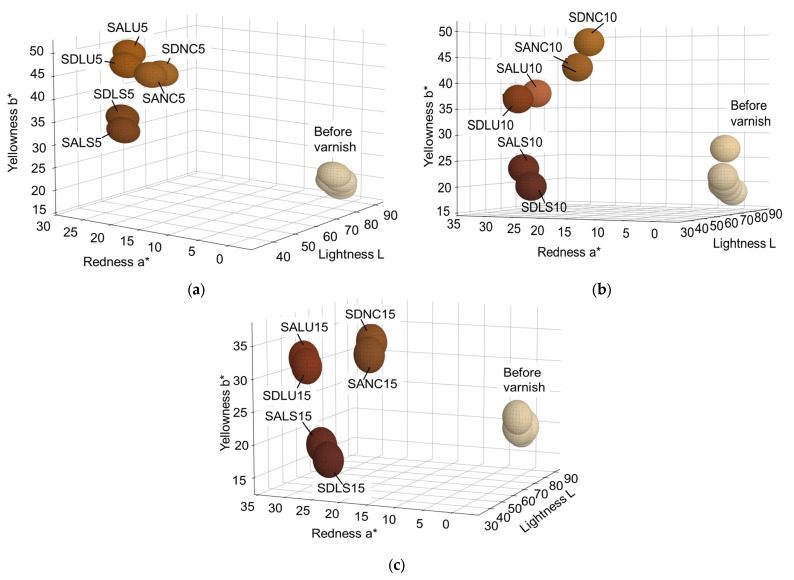
Distribution of varnished samples in the 3D Cie-Lab color space: (**a**) samples varnished with 5 layers; (**b**) samples varnished with 10 layers; (**c**) samples varnished with 15 layers.

Before and after varnishing, the samples were positioned in the 3D CIEL color space, according to Figure 9. It was found that the unvarnished samples form a cluster in terms of color parameters, regardless of the quality class of the spruce wood. After varnishing, the clusters formed depend on the type of varnish applied and the thickness of the layers. The largest dispersion of the clusters was visible for 15 layers of varnish (Figure 9c). The lowest values of lightness, redness and yellowness were in the case of samples varnished with spirit varnish, and the highest values of the color parameters are recorded for the samples varnished with nitrocellulose varnish, regardless of the film thickness (Figure 9). The proportion of late wood and the width of annual rings, according to which the quality class of spruce wood was established, were reflected in the color of the finish, regardless of the thickness of the applied layers. Of all the color parameters measured, the most consistent change was shown by redness, which increases on average from 1.57 to 23.73. Table 4 summarizes the average values of the color parameters. The mean and standard deviation were calculated for nine values from each sample category.

**Figure 10:**
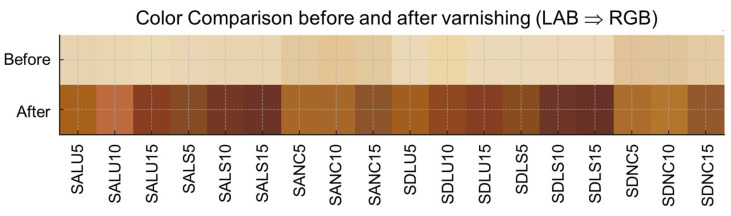
The color of samples in RGB space.

In Figure 10, the color correspondence of the measured parameters is presented, i.e., from the CIEL color space L*, a* and b* in the RGB color space (Red, Green and Blue), using a free application for conversion. In Table 5, the RGB values of the color parameters are summarized.

### 3.3. Surface Morphology of Varnished Wood Samples

**Figure 11:**
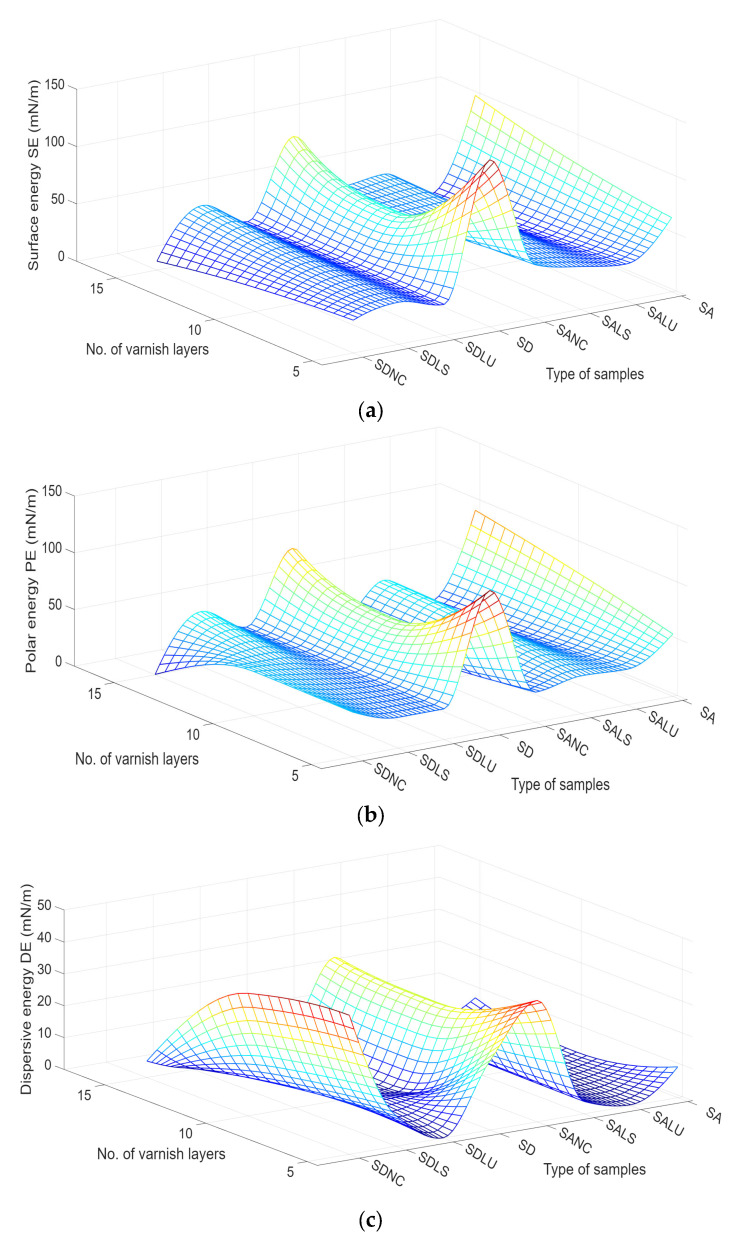
Variation in surface morphology in terms of (**a**) surface energy; (**b**) polar energy; and (**c**) dispersive energy. Legend: SA—unvarnished spruce wood grade A; SD—unvarnished spruce wood grade D.

In Figure 11, the average values of the surface energy of the polar component and of the dispersive component for the three types of varnishes, applied with different thicknesses, are presented comparatively. Unlike the varnished samples, the unvarnished spruce wood samples, denoted SA (wood grade A) and SD (wood grade D), have a higher degree of crystallinity, which leads to high values of the surface energy, compared to the energy of the varnished surfaces (Figure 11a). Of the three types of varnishes, the most hydrophobic varnished surface was that of oil-based varnish compared to nitrocellulose and alcohol varnishes. The surface energy is higher for wood samples of grade D, with a higher proportion of early wood. Analyzing the variation in surface energy only between the varnished samples, it is found that the lowest surface energy is presented by the oil-based varnish, approximately 30% lower than that of the NC and LS varnished samples (Figure 11a).

Also, the dispersion of the surface energy values was higher for the unvarnished wood because of the different anatomical characteristics and heterogeneity of the wood structure in comparison with the samples covered with varnish films. The two components of the surface energy (polar (Figure 11b) and dispersive (Figure 11c)) provide information about the polarity of the surface. Thus, the unvarnished wood presents a large polar component, larger than the dispersive one, indicating a polar (hydrophilic) surface. This hydrophilic behavior is also observed for the varnished samples, but the polarity of the surfaces was lower than for the unvarnished wood, regardless of the grade. With increasing varnishing film thickness, the polar energy decreases for the NC and LU varnishes, while for the LS varnished samples, the polarity increases by approximately 30%; this is in good agreement with the variation in AML, which registered the highest value for LS varnished samples attributable to its polar nature and large polar interactions throughout the hydrophilic bare wood surface. The dispersive component presented in Figure 11c was the result of physical dispersion forces; the polar one comes from stronger interactions between the component particles—chemical interactions, dipole–dipole and ion–dipole. The highest dispersive component is recorded for the samples coated with five layers of nitrocellulose varnish, and it decreases with the increasing number of layers. At the opposite pole, the lowest values of dispersive energy are recorded in samples varnished with oil-based varnish. The quality class of the wood covered with varnishes does not produce major changes in terms of the morphology of the varnished surfaces, as was highlighted by [33,37,38].

Celluloses are mainly responsible for the dissipative energy, while the matrix (lignin and hemicelluloses) is more sensitive to humidity variations and contributes to the viscoelasticity and swelling of the wood; therefore, it presents higher polar energy. Microfibrils and matrix are linked by hydrogen bonds and covalent cross-links, which impose hygroscopicity and dimensional instability in the material in general [10,39,40,41,42,43,44].

### 3.4. Correlation Between Color and Surface Parameters Based on Statistical Analysis

The following variables were used in the statistical analysis: type of wood substrate (denoted SS); type of varnish (TV); number of varnish layers (NL); areal mass loading (AML); color parameters before varnishing (lightness denoted LBV; redness aBV; yellowness bBV) and after varnishing (lightness denoted LAV; redness aAV; yellowness bAV); color difference (DE); surface energy (SE); polar component (PC); and dispersive component (DC). In the first stage, the influence of the three types of factors (wood quality class, varnish type and number of layers) on the determined physical and morphological properties was determined, the results being summarized in Table 6. With the exception of some color parameters (LBF, bBV, aAV and dE), most of the variables show a high level of variability (coefficients of variation: 20–139%), which implies the existence of sources for this variability. PC and DC are the properties with the highest dispersion of values around the mean (Table 6). None of the variables are normally distributed, so the course of non-parametric statistics was followed (Table 6).

**Figure 12:**
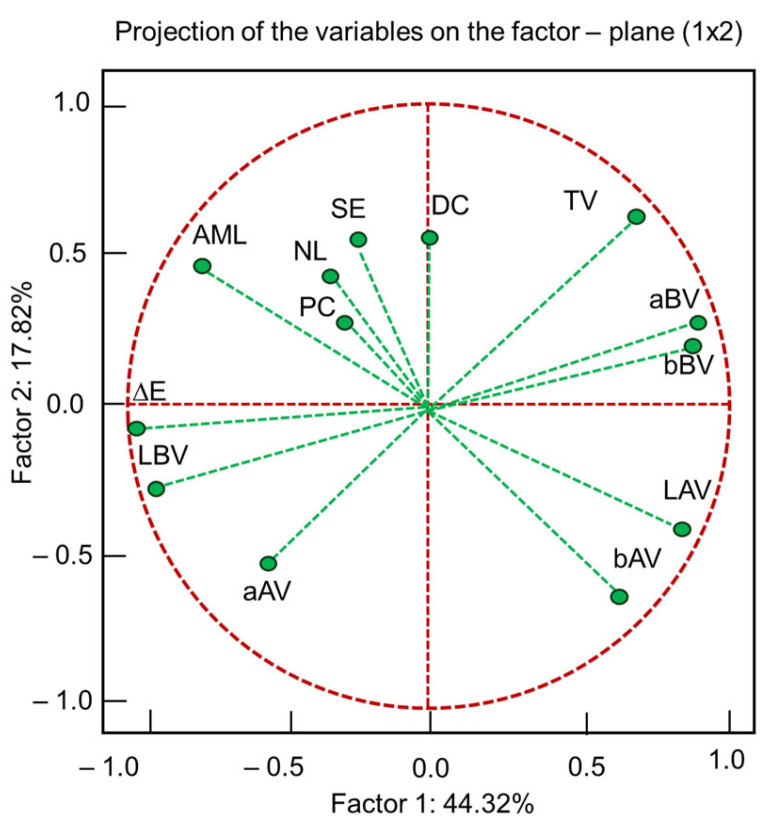
Principal component analysis (PCA).

AML, the color of the wood after varnishing, DC and SE are not influenced by the type of wood material (A, D). The color before varnishing varies depending on the type of wood (Table 6). PC also depends significantly on the type of wood. PC is the only property that is not influenced by the type of varnish used (Table 6). The multiple comparison matrix associated with the nonparametric significance test shows that the color differences before and after varnishing are significant only for nitrocellulose varnishes (*p* < 0.001; *p* = 0.26 for the other varnishes). Also, the differences in SE are due to the LU and NC varnishes (*p* < 0.03), and the differences in DC magnitude are only due to the LU varnish (*p* < 0.001). Among the properties studied, the number of varnish layers (NL) leaves its mark only on AML and the color of the wood after varnishing (Table 6). In contrast, SE, PC and DC have no relationship with the number of varnish layers applied. The change in the degree of redness of the color occurs only at five varnish layers, as shown by the multiple comparison matrix attached to the Kruskal–Wallis test. But the overall color differences (Δ*E*) change at each of the varnish layers. With PCA, five principal components were extracted, the first two together explaining 62% of the total variance, as can be seen in Figure 12. The first principal component, which explains 44% of the total variance, is oriented in the direction of the color difference Δ*E* (factor loadings = −0.99), which varies in tandem with the brightness of the color before varnishing. So, the lighter the wood, the more pronounced the effect of varnishing on the color. The second component is partly explained by the type of varnish (factor loadings = +0.63), which varies in tandem with DC and SE. The type of substrate is a non-influential variable.

According to the PCA, only the type of varnish and the number of varnish layers can be considered sources of variation for the analyzed properties.

### 3.5. FTIR Spectroscopy

**Figure 13:**
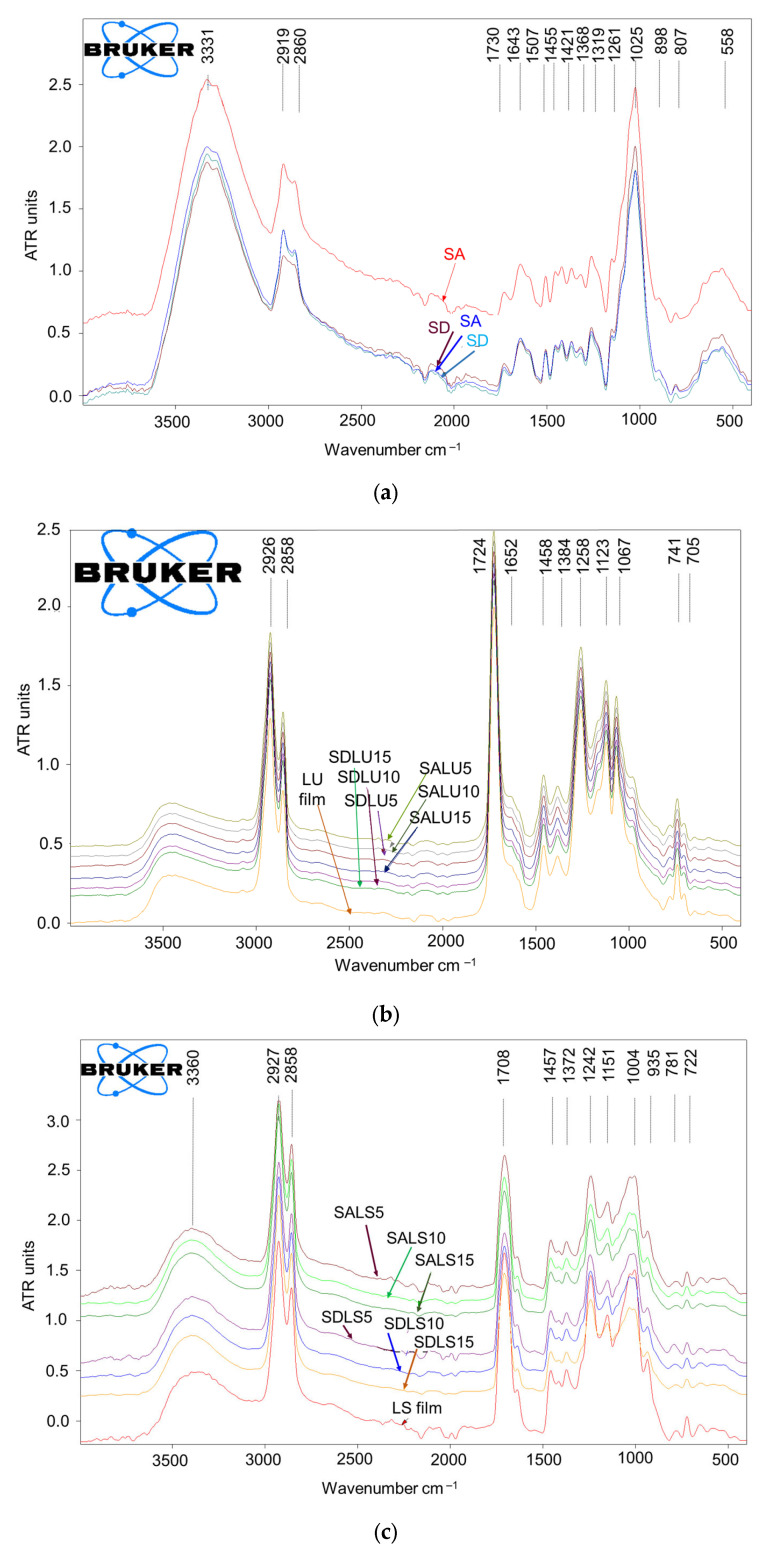

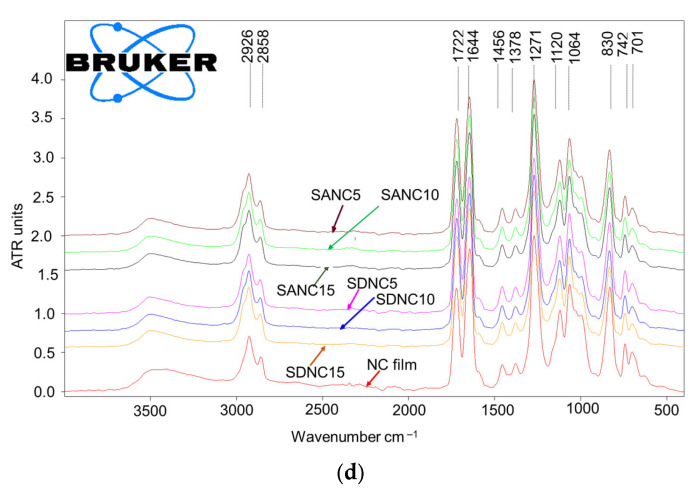
FTIR spectrum: (**a**) unvarnished samples—spruce wood (SA—spruce grade A, SD—spruce grade D); (**b**) wood samples with oil-based varnish; (**c**) samples with spirit varnish; (**d**) samples with nitrocellulose varnish.

In Figure 13, the FTIR spectra are presented for the spruce wood samples before varnishing (Figure 13a) and after varnishing with the three types of varnishes, each with the three categories of film thickness, resulting from 5, 10 and 15 layers of varnish (Figure 13b–d). Each type of varnish was also analyzed as a cured film on a glass slide for comparison. From the analysis of the presented spectra, for all types of varnishes (LU, LS, NC) and all types of wooden supports (SA, SD), it was found that they are like the spectra of the corresponding varnish films. This result denotes the fact that the wood structure was completely covered and was no longer visible in the FTIR spectra, not even in the case of the thinnest five-layer varnish finishing. For instance, specific absorption bands related to the main wood components, such as skeletal vibration of lignin (1507 cm^−1^), other lignin-related bands (region 1600–1640 cm^−1^, 1261 cm^−1^), C-H deformation vibration in cellulose and hemicelluloses (1368 cm^−1^) and C–O stretch vibrations in carbohydrates (1025 cm^−1^) were not visible at all in the spectra of varnished samples. At the same time, the absorption of hydroxyl groups (3300–3400 cm^−1^), present in large amounts in all the wood’s main components, was much receded, while the entire spectrum in the fingerprint region (1750–600 cm^−1^) was totally changed for the varnished samples compared to bare wood.

Higher film thicknesses (10 or 15 layers of varnish) or different classes of substrate (A, D) did not bring any change in terms of surface chemistry, the spectra being practically identical for a certain type of varnish for both class A and class D spruce wood and all film thicknesses. On the other hand, the spectra of the three types of varnishes were significantly different due to their different chemical compositions and structural features. It is important to say that even if a certain chemical feature (functional group, type of substructure or bond) is common to more compounds, the FTIR spectra will be different, with shifts of the respective absorption, depending on the whole chemical structural context of each compound. This is particularly visible in the fingerprint region of the FTIR spectra. Compared to unvarnished wood, for varnished wood, the most pronounced absorptions were the double band 2926–2858 cm^−1^, corresponding to aliphatic methylene and methyl groups (asymmetric and symmetrical C–H stretch), and the bands at 1724 cm^−1^ (LU), 1722 cm^−1^ (NC) and 1708 cm^−1^ (LS), all assigned to non-conjugated carbonyl groups in either carboxylic acids, esters aldehydes or ketones. These two features may indicate the presence of compounds with long aliphatic carbon chains and carbonyl groups, a good indication for coating materials with polymeric structures having unconjugated carbonyl groups in their chemical structure. For the samples varnished with nitrocellulose, a high absorption band at 1644 cm^−1^, specific to the –NO_2_ group associated with a methylene—CH_2_– group (bending) in nitrocellulose, was highlighted. This is different from the moderate, not well-defined absorption at 1643 cm^−1^, visible in the spectra of unvarnished wood, associated with conjugated and aromatic carbonyl groups, which might be present in the structure of lignin [41,42,43,44]. In the next range of the spectra, there were noted close peaks at 1271 cm^−1^ (NC), 1258 cm^−1^ (LU), and 1242 cm^−1^ (LS) for the three types of varnishes, which can be associated with specific structural features of these materials as follows: symmetrical stretch of –NO2 (valence vibration) for samples varnished with nitrocellulose varnish; C–O–C (stretching) of oxirane groups for oil-based varnish; and O–H (bending) and C–O (stretching) in hydroxyl –OH, carboxyl –COOH, and ester groups for alcoholic varnish LS. In unvarnished wood, the peak at 1025 cm^−1^, assigned to C–O vibration in ethers and alcohols, is associated with cellulose and hemicelluloses; for the varnished samples, similar vibrations were observed at slightly different wavenumbers: 1004 cm^−1^ (LS), highlighting C–O (stretching) in alcohols, ether bonds, and acetals; 1065 cm^−1^ (NC), highlighting C–O (stretching) in the pyranose ring in nitrocellulose; and 1067 cm^−1^ (LU) C–O (stretching) of primary alcohols, highlighting the chemical structure particularities of each film-forming resin.

## 4. Conclusions

This study presents the surface changes in terms of color, morphology and chemical fingerprint of spruce wood varnished with three types of varnishes (polymeric and alcohol solvent). The research highlighted the following aspects:

- The color parameters depend on the type of varnish and the thickness of the varnish film; the largest changes in redness and yellowness are recorded when applying the five layers containing the coloring pigments.
- The fastest stabilization of color parameters was observed with nitrocellulose varnish.
- Spruce wood with wide annual rings and a higher proportion of early wood (quality class D) has 25% higher surface energy compared to spruce wood with narrow annual rings (class A).
- The varnish film modifies the surface energy of the wood, reducing it by approximately 50% compared to unvarnished wood, the lowest values being recorded for oil-based varnish.
- Polar and dispersive energy depend on the varnish solvent and the number of layers applied.
- The varnish film modifies the FTIR absorption bands, completely covering the wood, leading to the formation of a new layered material consisting of the wood–varnish interface and the varnish film.

Future studies are needed to determine which varnish recipe offers the best viscoelastic properties of soundboard for dynamics in musical instrument construction.

## Figures and Tables

**Table 1:** Materials for finishing.

Type of Material (Code)	Trade Name	Producer	Website
Varnishes			
Oil-based varnish (LU)	Joha Oil Varnish Standard	Hammerl GmbH & Co. KG	https://www.hammerl.com/en/varnishes.html (accessed on 27 July 2025)
Alcohol varnish (LS)	Joha Spirit varnish standard	Hammerl GmbH & Co. KG	https://www.hammerl.com/en/varnishes.html (accessed on 27 July 2025)
Nitrocellulose varnish (NC)			
Coloring materials			
Color extracts for varnishes (CEV)	Joha red (2903) Joha yellow (2901) Joha brown (2904)	Hammerl GmbH & Co. KG	https://www.hammerl.com/en/varnishes.html (accessed on 27 July 2025)
Water stains * (WS)	169 red brown 125 pure yellow 104 nut brown dark	Hammerl GmbH & Co. KG	https://www.hammerl.com/en/varnishes.html (accessed on 27 July 2025)
Kalium dichromate ** (KD)	-	International Laboratory SRL, Cluj Napoca, Romania	

Notes: * WS-R recipe: nut brown dark 5 g, pure yellow 2.5 g, and red brown 2.5 g in 1000 mL water for samples varnished with LU; ** employed as water solution: Kalium dichromate 53.5 g/1000 mL water.

**Table 3:** The average values of AML and the increase.

Samples	AML (kg/m^2^)	∆AML (%)
SALU5	0.068	-
SALU10	0.090	33.052
SALU15	0.129	42.580
SALS5	0.167	-
SALS10	0.183	9.606
SALS15	0.200	9.409
SANC5	0.036	-
SANC10	0.068	90.322
SANC15	0.106	55.205
SDLU5	0.076	-
SDLU10	0.097	28.732
SDLU15	0.134	37.949
SDLS5	0.149	-
SDLS10	0.173	16.624
SDLS15	0.223	28.498
SDNC5	0.044	-
SDNC10	0.084	90.844
SDNC15	0.131	56.573

**Table 4:** The average and standard deviation values of color parameters.

Samples	Before Varnish			After Varnish			
	L*	a*	b*	L*	a*	b*	∆E*
SALU5	85.49 (0.86)	2.06 (0.50)	19.46 (0.62)	48.37 (3.44)	24.03 (1.76)	49.13 (2.39)	52.34
SALU10	86.13 (0.17)	1.74 (0.22)	20.02 (0.64)	39.43 (4.62)	29.43(0.54)	38.06 (7.35)	46.19
SALU15	87.12 (0.25)	1.29 (0.17)	19.54 (0.47)	35.86 (2.02)	30.38 (0.47)	32.39 (3.05)	60.32
SALS5	86.40 (0.42)	1.58 (0.07)	19.33 (0.40)	38.42 (1.21)	21.08 (3.76)	33.79 (2.23)	53.77
SALS10	86.11 (1.14)	1.96 (0.46)	19.61 (0.69)	31.35 (2.63)	25.43 (0.90)	23.55 (4.33)	59.90
SALS15	85.99 (0.22)	1.83 (0.10)	19.51 (0.25)	29.02 (0.41)	25.44 (0.59)	19.92 (1.24)	61.68
SANC5	85.01 (0.62)	1.64 (0.36)	21.14 (0.50)	49.78 (0.85)	20.80 (0.20)	44.57 (1.42)	46.45
SANC10	84.72 (1.11)	1.77 (0.97)	21.15 (0.91)	49.92 (1.41)	20.54 (0.40)	43.20 (1.43)	45.27
SANC15	84.94 (0.30)	1.64 (0.27)	21.27 (0.76)	41.85 (1.08)	19.80 (0.53)	33.13 (1.29)	48.24
SDLU5	87.38 (0.63)	1.38 (0.27)	18.70 (0.90)	46.74 (3.66)	23.92 (1.40)	46.57 (5.39)	54.19
SDLU10	86.34 (1.16)	1.68 (0.33)	18.96 (1.59)	38.75 (3.77)	28.66 (3.43)	36.97 (6.72)	55.63
SDLU15	87.16 (0.28)	1.40 (0.15)	19.06 (1.10)	35.44 (4.71)	29.80 (3.04)	31.31 (7.69)	60.26
SDLS5	87.22 (0.29)	1.48 (0.25)	18.08 (1.27)	39.08 (3.20)	21.47 (3.16)	36.40 (3.26)	55.26
SDLS10	87.06 (0.31)	1.54 (0.13)	18.93 (0.69)	29.44 (2.55)	23.58 (1.39)	20.24 (5.35)	61.71
SDLS15	87.05 (0.28)	1.41 (0.15)	19.07 (0.26)	27.74 (1.23)	23.93 (2.03)	17.92 (3.26)	63.45
SDNC5	86.68 (0.42)	1.15 (0.65)	19.79 (1.27)	51.80 (1.22)	19.63 (0.58)	44.83 (3.34)	46.75
SDNC10	87.85 (1.24)	0.71 (0.54)	18.10 (0.55)	54.61 (2.83)	19.61 (0.77)	48.22 (1.43)	48.68
SDNC15	87.24 (0.62)	0.86 (0.32)	19.76 (0.90)	43.36 (1.03)	19.71 (0.68)	35.02 (1.88)	50.14

**Table 5:** The RGB color parameters.

Samples	Before Varnish			After Varnish		
	Red	Green	Blue	Red	Green	Blue
SALU5	232	211	177	169	97	27
SALU10	234	213	178	148	71	30
SALU15	235	216	181	138	62	33
SALS5	234	214	180	134	76	35
SALS10	234	213	179	118	56	38
SALS15	233	213	178	111	51	34
SANC5	231	210	173	168	103	40
SANC10	230	209	172	168	104	44
SANC15	231	210	172	142	85	44
SDLU5	236	217	184	164	93	29
SDLU10	233	214	180	145	70	31
SDLU15	235	216	182	136	61	34
SDLS5	235	216	184	137	77	32
SDLS10	235	216	182	110	53	39
SDLS15	235	216	182	105	49	39
SDNC5	234	215	180	172	109	45
SDNC10	235	219	186	181	116	44
SDNC15	235	217	181	146	89	44

**Table 6:** Sources of variation in the properties of studied samples.

Sample Properties	Mean	Standard Deviation	Normality *	The Significance of Differences Between		
				SS **	TV ***	NL ***
AML	0.126	0.05	NO	0.6800	*<0.001*	*<0.001*
LBF	84.903	2.63	NO	*0.0020*	-	
aBF	2.492	1.42	NO	*0.0300*	-	
bBV	21.151	3.01	NO	*0.0010*		
LAV	40.039	8.11	NO	0.5000	*<0.001*	*<0.001*
aAV	23.946	3.93	NO	0.3700	*<0.001*	*<0.001*
bAV	34.691	9.90	NO	0.6500	*<0.001*	*<0.001*
DE	52.752	9.04	NO	0.1900	*<0.001*	*<0.001*
SE	51.263	39.38	NO	0.6800	*<0.001*	0.87
PC	35.075	35.96	NO	*0.0200*	0.28	0.13
DC	16.234	22.63	NO	0.1300	*<0.001*	0.4

* The hypothesis of normality of the distribution of variables was verified with the Shapiro–Wilk test: YES—the hypothesis of normality cannot be rejected/NO—the hypothesis of normality cannot be accepted. ** *p* from Mann–Whitney U test. *** *p* from Kruskal–Wallis test. Values highlighted in italics are significant for the statistical test used.

## Data Availability

The original contributions presented in this study are included in the article. Further inquiries can be directed to the corresponding author.
